# Effect of White Potatoes on Subjective Appetite, Food Intake, and Glycemic Response in Healthy Older Adults

**DOI:** 10.3390/nu12092606

**Published:** 2020-08-27

**Authors:** Nick Bellissimo, Robena Amalraj, Jennifer J. Lee, Neil R. Brett, Julia O. Totosy de Zepetnek, Sarah Proteau, Dérick Rousseau

**Affiliations:** 1 School of Nutrition, Faculty of Community Services, Ryerson University, 350 Victoria Street, Toronto, ON M5B 2K3, Canada; robena.amalraj@ryerson.ca (R.A.); j585lee@ryerson.ca (J.J.L.); neil.brett@ryerson.ca (N.R.B.); proteausarah06@gmail.com (S.P.); 2 Faculty of Kinesiology and Health Studies, University of Regina, 3737 Wascana Parkway, Regina, SK S4S 0A2, Canada; julia.totosy@uregina.ca; 3 Department of Chemistry and Biology, Faculty of Science, Ryerson University, 350 Victoria Street, Toronto, ON M5B 2K3, Canada; rousseau@ryerson.ca

**Keywords:** white potatoes, older adults, elderly, appetite, food intake, glycemia

## Abstract

The objective of this study was to determine the effect of white potato cooking methods on subjective appetite, short-term food intake (FI), and glycemic response in healthy older adults. Using a within-subject, repeated-measures design, 20 participants (age: 70.4 ± 0.6 y) completed, in random order, five treatment conditions: three potato treatments (baked potatoes, mashed potatoes, and French fries), an isocaloric control treatment (white bread), or a fasting condition (meal skipping). Subjective appetite and glycemic response were measured for 120 min using visual analogue scales and capillary blood samples, respectively. Lunch FI was measured with an ad libitum pizza meal at 120 min. Change from baseline subjective appetite (*p* < 0.001) and lunch FI (*p* < 0.001) were lower after all test treatments compared with meal skipping (*p* < 0.001), but did not differ among test treatments. Cumulative FI (test treatment + lunch FI) did not differ among treatment conditions. Blood glucose concentrations were higher after all test treatments compared with meal skipping (*p* < 0.001), but were not different from each other. In healthy older adults, white potatoes suppressed subjective appetite and lunch FI compared with meal skipping, suggesting white potatoes do not bypass regulatory control mechanisms of FI.

## 1. Introduction

The number of older adults in the United States [1] and Canada [2] is expected to increase more rapidly than other age groups in the next few decades. Older adults are at a higher risk of adverse health outcomes associated with suboptimal dietary intake compared with young adults, in part, due to age-related changes in physiological, metabolic, and hormonal factors, which can also affect components of energy balance [3,4,5,6]. Aging is associated with elevated satiety, diminished food intake (FI), incomplete caloric compensation, and impaired glycemic regulation [6,7], which likely contribute to the high prevalence of metabolic disorders associated with both overnutrition (e.g., obesity [8], type 2 diabetes [9]) and undernutrition (e.g., malnutrition) [3,4,10], the two opposing consequences of energy balance dysregulation.

White potatoes provide energy and contribute vitamins, minerals, and fiber to the diet [11]. Although some epidemiologic studies have shown associations between white potato consumption and weight gain [12], type 2 diabetes [13], and mortality [14], recent experimental studies are discordant with the foregoing observations. For example, daily consumption of non-fried potatoes for four weeks in adults did not have an adverse effect on blood glucose, body weight, or cardiometabolic markers [15]. Other experimental studies have shown white potatoes increase satiety and lower FI in children [16,17] and young adults [18,19]. In one experimental study examining the effects of potatoes in older adults, higher satiety was found after consuming 50 g of available carbohydrates from mashed potatoes compared with barley and a glucose solution, and FI 2 h later was lower after mashed potatoes and barley compared with the glucose solution and the control [20]. However, it is not clear how the cooking method of white potatoes affects satiety, FI, or glycemic response in older adults. Therefore, the objective of the present study was to compare the effects of different white potato cooking methods on subjective appetite, short-term FI, and glycemic response in healthy older adults.

## 2. Materials and Methods

### 2.1. Participants

Non-smoking older adults, defined as 65 years of age and over [21], were recruited via word-of-mouth, advertisements in local newspapers, and social media. Inclusion criteria were being within the healthy body mass index (BMI) range for older adults of 18.5–29.9 kg/m^2^ [22,23], not taking any medications that could affect appetite or FI, and regularly consuming breakfast, defined as eating any caloric food or drinks within 2–4 h of awakening at least 5 days a week [24]. Exclusion criteria were individuals who had a previous diagnosis of diabetes, pre-diabetes, gastrointestinal disease, liver disease, kidney disease, or sensory impairments; had a major medical or surgical event within the past 6 months; are or were on a diet within the past 6 months; or had food allergies, intolerances, sensitivities, dislikes, and/or dietary restrictions to any of the foods used in the study. An initial telephone interview was conducted to determine eligibility, and eligible participants were scheduled for an in-person information session to obtain informed written consent.

### 2.2. Experimental Design

During the in-person information session, fasted (>8 h) blood glucose levels were assessed using a finger-prick capillary blood sample. Participants were excluded if they had impaired fasting blood glucose as defined by elevated fasting glucose concentration of ≥5.6 mmol/L [25]. Participant’s height was measured and recorded to the nearest 0.1 cm (SECA 220 height rod, SECA Group, Hamburg, Germany) and body mass was measured to the nearest 0.1 kg (Bod Pod, Life Measurement Instruments, Concord, CA, USA). Body composition, fat mass (%) and fat-free mass (%), was estimated from the Bod Pod [26] using sex- and ethnic-specific body fat equations for adults [27]. Participants were familiarized with the test day procedures including visual analogue scales (VAS) and the finger-prick blood collection procedures. Participants rated two pizza preferences from three varieties: three-cheese, pepperoni, or deluxe (Dr. Oetker, Giuseppe Pizzeria Mini Pizzas, Mississauga, ON, Canada). The study protocol was approved by the Ryerson University Research Ethics Board and registered at ClinicalTrials.gov (NCT03309124).

Following the familiarization session, participants completed five treatment conditions using a randomized, within-subject, repeated-measures design. Participants arrived at the lab between 8:00 and 10:00 am (time of arrival remained consistent for each participant throughout the study period) on five separate mornings, approximately one week apart, following a 12 h overnight fast (water was permitted up to 1 h before arrival). Upon arrival, participants were asked if they consumed anything to eat or drink (other than water), whether they consumed any medications, the type and intensity of physical activity in the previous 24 h, and sleep duration. If there were any deviations from their normal routine or if baseline blood glucose levels were ≥5.6 mmol/L, the session was rescheduled. After baseline measures were collected, participants consumed one of four test treatments (baked potatoes, mashed potatoes, French fries, or white bread) within 15 min or continued to fast (i.e., meal skipping). Subjective appetite, subjective emotions, subjective physical well-being, and glycemic response were measured at baseline (0 min), and 15, 30, 45, 60, 90, and 120 min post-treatment consumption. Participants consumed an ad libitum pizza lunch at 120 min to assess lunch FI.

#### Test Treatments

To examine the effect of one serving of the different white potato products, four caloric test treatments were matched for total fat (13.7 g) content and available carbohydrate (33.1 g) content, equivalent to one baked medium russet potato as per the United States Department of Agriculture (USDA) Nutrient Database [28]. Three white potato products were prepared using different methods: (1) oven-baked (baked potatoes), (2) boiled, mashed, frozen, then reheated in a microwave (mashed potatoes), and (3) deep-fried in oil (French fries); these methods were compared with the calorically-matched control treatment (white bread as recommended by Health Canada [29]) and non-caloric control condition (meal skipping). Salt was added to French fries and baked potatoes to match the sodium content (284 mg) to white bread, while enhancing palatability as determined by a priori taste panel. Treatment composition and characteristics are shown in Table 1.

All test treatments were prepared in a laboratory kitchen according to a standardized protocol and were served warm with ad libitum water (Nestlé Pure Life^®^, Toronto, ON, Canada). All potato treatments were prepared with the skin. Baked potatoes were prepared by oven-baking a russet potato in a conventional oven and adding canola oil (Saporito Foods Inc., Markham, ON, Canada) and salt (0.6 g). Mashed potatoes were prepared from fresh russet potatoes, seasoned, and subsequently frozen by the manufacturer (Lightly Seasoned Boiled Mashed Potatoes, donated by McCain Foods Limited, Canada). Frozen mashed potatoes were microwaved in the laboratory kitchen and mixed with canola oil. French fries were prepared from frozen potato strips (Straight Cut French Fries, donated by McCain Foods Limited, Canada), deep-fried in canola oil, and served with salt (0.6 g) on top. White bread (Wonder Bread, Weston Bakeries Limited, Kitchener, ON, Canada) was toasted in a toaster oven and canola oil was added before serving. The total energy and macronutrient composition of baked potatoes, mashed potatoes, and white bread were calculated based on the USDA’s Nutrient Database and nutrition information provided by the manufacturer. The macronutrient composition of French fries was determined by Maxxam Analytics Inc. (Mississauga, ON, Canada) using the Association of Official Analytical Chemists (AOAC) methods 922.06 (acid hydrolysis method for fat analysis), 933.05 (modified Mojonnier ether extraction method for fat analysis), and 992.15 (combustion method for protein analysis) [30] to account for any changes in the nutrient content following deep-frying in oil. Baked potatoes provided 288 kcal with 4.6 g of protein and 37.1 g of carbohydrates (4.0 g of fiber). Mashed potatoes provided 283 kcal with 3.3 g of protein, 36.4 g of carbohydrates (3.3 g of fiber), and 579 mg of sodium. French fries provided 280 kcal with 3.0 g of protein and 36.2 g of carbohydrates (3.1 g of fiber). White bread provided 280 kcal with 5.7 g of protein and 35.0 g of carbohydrates (1.9 g of fiber).

### 2.3. Experimental Procedures

#### 2.3.1. Subjective Appetite and Emotions

The effect of treatment on subjective appetite, emotions, and physical well-being were assessed using a 100-unit line VAS questionnaire, administered using a computerized application (Express VAS, Toronto, ON, Canada) on an ASUS laptop (ASUS Transformer Book 10.1” Touch Convertible Laptop, ASUS, Taipei, Taiwan). Participants were instructed to place an ‘X’ along the 100-unit line between two opposing statements (e.g., ‘not at all’ and ‘very’) at each time point depending on how they felt at the moment. Scores were determined by measuring the distance (units) from the left starting point to the ‘X.’

Subjective appetite was determined using a motivation-to-eat VAS questionnaire consisting of four questions: (1) desire-to-eat question of ‘how strong is your desire to eat?’ (‘very weak’ to ‘very strong’); (2) hunger question of ‘how hungry do you feel?’ (‘not hungry at all’ to ‘very hungry’); (3) fullness question of ‘how full do you feel?’ (‘not full at all’ to ‘very full’); and (4) prospective food consumption question of ‘how much food do you think you can eat?’ (‘nothing at all’ to ‘a large amount’) [31,32,33]. Subjective average appetite scores (units) were calculated from the four VAS question scores as follows, as previously reported [17,31,32,33,34]: (desire-to-eat + hunger + (100-fullness) + prospective food consumption)/4.

Subjective emotions were determined using an emotion VAS questionnaire consisting of eleven questions, as previously reported [34,35]: (1) how alert do you feel right now? (‘not alert at all’ to ‘very alert’); (2) how happy do you feel right now? (‘not happy at all’ to ‘very happy’); (3) how excited do you feel right now? (‘not excited at all’ to ‘very excited’); (4) how sad do you feel right now? (‘not sad at all’ to ‘very sad’); (5) how tense do you feel right now? (‘not tense at all’ to ‘very tense’); (6) how sleepy do you feel right now? (‘not sleepy at all’ to ‘very sleepy’); (7) how exhausted do you feel right now? (‘not exhausted at all’ to ‘very exhausted’); (8) how aggressive do you feel right now? (‘not aggressive at all’ to ‘very aggressive’); (9) how angry do you feel right now? (‘not angry at all’ to ‘very angry’); (10) how disappointed do you feel right now? (‘not disappointed at all’ to ‘very disappointed’); and (11) how frustrated do you feel right now? (‘not frustrated at all’ to ‘very frustrated’). An average subjective emotion score (unit) was calculated from the eleven VAS question scores using the following equation: (alertness + happiness + excitement + 800—(sadness + tenseness + sleepiness + exhaustion + aggression + anger + disappointment + frustration))/11. Physical well-being was assessed using a wellness VAS question ‘how well do you feel right now?’ with anchors ‘not well at all’ to ‘very well.’ All VAS questionnaires were administered at baseline (0 min), and 15, 30, 45, 60, 90, and 120 min post-treatment consumption. Pleasantness of the test treatment was assessed using a VAS question of ‘how pleasant did you find the meal?’ with anchors ‘not pleasant at all’ to ‘very pleasant’ at 15 min post-treatment consumption.

#### 2.3.2. Food and Water Intake

Lunch FI was measured at 120 min with an ad libitum pizza lunch, as reported in our previous studies [17,31,32,33]. Participants were escorted to a sensory room and individually seated in a semi-private cubicle with ad libitum water for 30 min. A warm tray of four mini pizzas, three pizzas of participants’ first choice and one mini pizza of their second choice, was served every 10 min. Participants were instructed to eat until they were comfortably full. The weight of the leftover pizza (g) was deducted from the served pizza weight to determine the weight of the consumed pizza, then consumed energy (kcal) was calculated using nutrition information on the nutrition facts panel provided by the manufacturer. Each frozen pepperoni pizza provided approximately 200 kcal and weighed 87 g with 9 g of protein, 27 g of carbohydrates (1 g of fiber), and 7 g of fat. Each frozen deluxe pizza provided approximately 200 kcal and weighed 92 g with 8 g of protein, 27 g of carbohydrates (1 g of fiber), and 6 g of fat. Each frozen three-cheese pizza provided approximately 200 kcal and weighed 82 g with 9 g of protein, 27 g of carbohydrates (1 g of fiber), and 6 g of fat. On average, each pizza provided 17% of energy as protein, 52% as carbohydrates, and 29% as fat. The pizzas were selected due to lack of an outer crust to provide consistent energy density and macronutrient distribution, as previously reported [36]. Water was weighed before and after the pizza lunch to determine water consumption (g). Cumulative FI (kcal) was calculated by adding the energy from the test treatment and ad libitum pizza lunch.

#### 2.3.3. Blood Glucose

Capillary blood samples were collected using a single-use, auto-disabling finger-prick lancet device (SLB200, Surgilance^TM^, Brussels, Belgium) in sodium fluoride/potassium oxalate capillary blood collection tubes (Terumo America Inc., CA, USA), as previously reported [33,34]. Blood samples were collected at baseline (0 min), and at 15, 30, 45, 60, 90, and 120 min post-treatment consumption. Whole blood samples were analyzed immediately for blood glucose using a YSI 2300 Stat Plus Glucose Analyzer (YSI Inc., Yellow Springs, OH, USA).

### 2.4. Statistical Analyses

SAS version 9.4 (SAS Institute Inc., Carey, NC, USA) was used for all statistical analyses with statistical significance considered at *p* < 0.05. With the PROC MIXED model, repeated-measures two-factor analysis of variance (ANOVA) was used to determine the effect of treatment, time, and treatment by time interaction on change from baseline average appetite scores, mean emotion VAS scores, physical well-being scores, and blood glucose concentrations. Subjective average appetite incremental area under the curve (iAUC) and blood glucose iAUC were calculated using the trapezoid method [37]. The effect of treatment on average appetite iAUC, blood glucose iAUC, lunch FI, and cumulative FI were analyzed using a one-factor repeated-measures ANOVA. Tukey–Kramer’s post hoc analyses to account for multiple comparisons were performed when the main effects of treatment by time interactions were observed. Pearson’s correlation coefficients were used to examine the associations between subjective appetite iAUC, lunch FI, and blood glucose iAUC for each test treatment and pooled data. All data are presented as means ± standard error of the mean (SEM).

## 3. Results

Twenty-two adults over 65 y completed the study; however, two participants were removed from analyses due to an outlier FI data <500 kcal at lunch on the control visit and non-compliance to the study protocol (i.e., reported restrained eating at the ad libitum pizza lunch). The final sample size for data analyses was twenty (7 males and 13 females). Baseline characteristics are summarized in Table 2.

### 3.1. Subjective Appetite and Emotions

**Figure 1:**
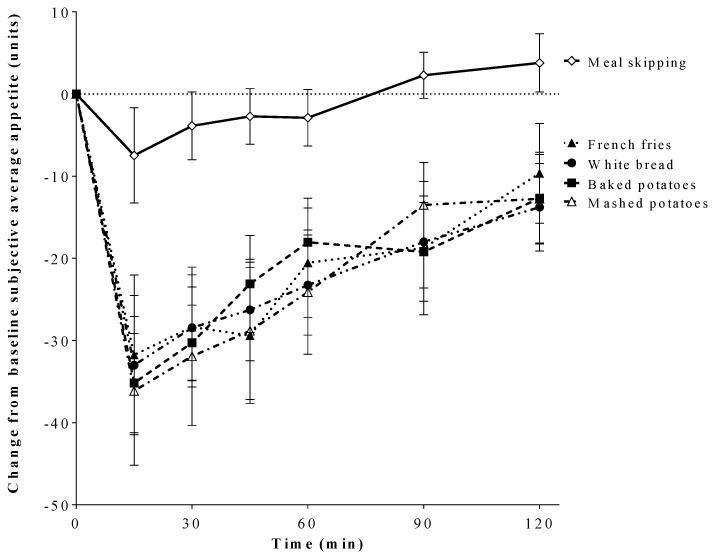
Effect of treatment on change from baseline average appetite scores (units) over 120 min (two-factor ANOVA, Tukey-Kramer’s post hoc test). All values are means ± SEM, *n* = 20. Change from baseline subjective appetite was affected by treatment (*p* < 0.001), time (*p* < 0.001), and treatment by time interaction (*p* < 0.001). Change from baseline subjective appetite was lower after all caloric test treatments compared with meal skipping (*p* < 0.001), but did not differ from each other.

Change from baseline subjective average appetite scores were affected by treatment (*p* < 0.001), time (*p* < 0.001), and treatment by time interaction (*p* < 0.001). Change from baseline subjective appetite scores were lower after all test treatments compared with meal skipping, but did not differ among test treatments (Figure 1). Subjective appetite iAUC (units • min) was affected by treatment (*p* < 0.001). Subjective appetite iAUC was lower after baked potatoes (−1928.2 ± 277), mashed potatoes (−2396.6 ± 426), French fries (−2058.7 ± 376), and white bread (−1637.2 ± 344) compared with meal skipping (−278.4 ± 304; *p* < 0.001); however, there was no difference among test treatments.

Change from baseline average subjective emotions and physical well-being scores were not affected by treatment (*p* > 0.1), time (*p* > 0.05), and there was no significant treatment by time interaction (*p* > 0.5). Test treatment pleasantness was affected by treatment (*p* < 0.001), where participants reported baked potatoes (*p* = 0.03) and French fries (*p* = 0.001) to be more pleasant than white bread (Table 3).

### 3.2. Food and Water Intake

There was a main effect of treatment on lunch FI (*p* < 0.001). Lunch FI was lower after baked potatoes (*p* = 0.001), mashed potatoes (*p* = 0.01), French fries (*p* = 0.004), and white bread (*p* = 0.01) compared with meal skipping, but not different among test treatments. Cumulative FI (test treatment + lunch FI) was not affected by treatment (*p* = 0.26). Neither water intake with test treatment (*p* = 0.60) nor with ad libitum pizza lunch (*p* = 0.27) was affected by treatment (Table 3).

### 3.3. Blood Glucose

**Figure 2:**
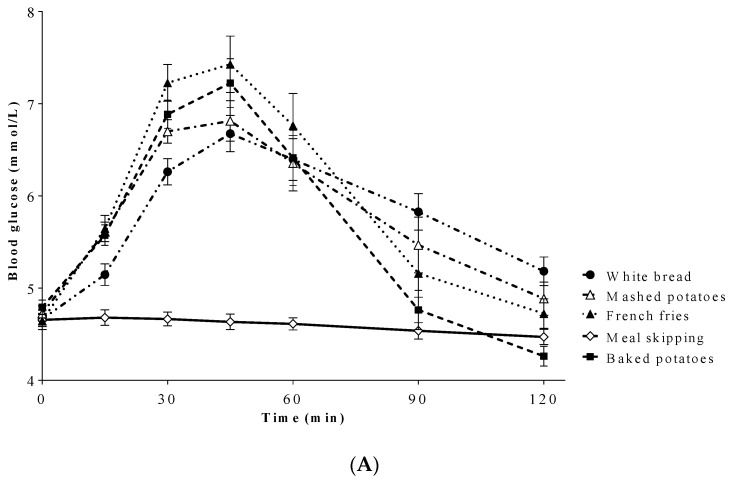

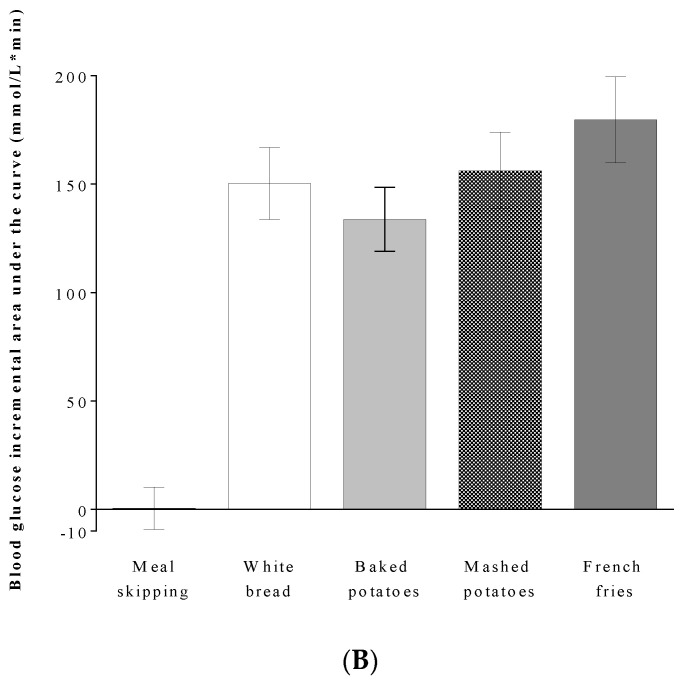
(**A**) Effect of treatment on blood glucose over 120 min. Blood glucose concentrations were affected by treatment (*p* < 0.001), time (*p* < 0.001), and treatment by time interaction (*p* < 0.001). Blood glucose concentrations were higher after all test treatments compared with meal skipping (*p* < 0.001), but did not differ from each other. (**B**) Effect of treatment on blood glucose incremental area under the curve (iAUC). Blood glucose iAUC was affected by treatment (*p* < 0.001), where iAUCs were higher after all test treatments compared with meal skipping. All values are means ± SEM, *n* = 20.

Blood glucose concentrations were affected by treatment (*p* < 0.001), time (*p* < 0.001), and treatment by time interaction (*p* < 0.001). Blood glucose concentrations were higher after baked potatoes (*p* < 0.001), mashed potatoes (*p* < 0.001), French fries (*p* < 0.001), and white bread (*p* < 0.001) compared with meal skipping, but did not differ from each other. Blood glucose concentrations were higher after French fries compared with white bread at 30 min (*p* = 0.03), and lower after baked potatoes compared with white bread at 90 min (Figure 2A). There was a main effect of treatment on blood glucose iAUC (*p* < 0.001). Blood glucose iAUC (mmol/L • min) was higher after baked potatoes (133.7 ± 14.7), mashed potatoes (156.2 ± 17.7), French fries (179.6 ± 19.8), and white bread (150.2 ± 16.5) compared with meal skipping (0.53 ± 9.8; *p* < 0.001); however, there was no difference among test treatments (Figure 2B).

### 3.4. Correlations

There were no significant associations among subjective appetite iAUC, lunch FI, and blood glucose iAUC after any of the individual test treatments or in the pooled samples.

## 4. Discussion

The objective of the present study was to examine the effects of different white potato cooking methods on subjective appetite, short-term FI, and glycemic response in healthy older adults (≥ 65 y). The main finding was that all test treatments decreased subjective appetite scores and lunch FI compared with meal skipping; however, cumulative FI was not different compared with meal skipping. Although the glycemic response was higher after all test treatments compared with meal skipping, the test treatments were not significantly different from each other.

Similar to previous studies, all white potato treatments suppressed appetite and lunch FI compared with meal skipping in older adults. White potatoes have been shown to exert higher feelings of satiety and/or lower FI compared with other carbohydrate-containing foods matched for available carbohydrates in older adults [20] and other age groups, including young adults [19,38] and children [17,34]. In young adults, mashed potatoes resulted in higher satiety ratings over 120 min compared with other carbohydrate and protein foods matched for available carbohydrate content [38]. In children, white potatoes consumed with a fixed portion of protein food lowered subjective appetite and reduced FI at 180 min post-treatment compared with an isocaloric meal of cereal and white bread and meal skipping [17]. Potato protease inhibitor II in potatoes may suppress appetite and reduce FI [39] by inhibiting cholecystokinin (CCK)-degrading enzymes [40,41], which can increase serum CCK to delay stomach motility [42]. Furthermore, CCK may play a more significant role in satiety in older compared with young adults due to higher fasting and post-prandial CCK concentrations in older adults [43]. Future studies measuring CCK and other gastrointestinal hormones are warranted to examine the physiological mechanism of white potatoes on FI regulation.

Cumulative FI after all test treatments did not differ from meal skipping, suggesting older adults compensate for previously skipped calories at the next meal. In contrast to our findings, a study in older adults showed similar cumulative FI over 120 min after mashed potatoes, barley, glucose, and the control, but mashed potatoes had higher caloric compensation scores compared with glucose [20]. It has been proposed that older adults generally consume less food [3], have lower fasting hunger levels, and increased satiation [44] compared with young adults, often referred to as the ‘anorexia of aging.’ Our findings showed no evidence for age-related declines in habitual motivation-to-eat in healthy older adults, as cumulative FI was similar compared with studies in children [17] and adults [18,19] employing similar study designs. However, undernourished older adults may show more pronounced alterations in habitual motivation-to-eat and energy compensation compared with well-nourished older adults due to the manifestation of the anorexia of aging. Undernourished older women showed lower baseline hunger levels and higher caloric compensation following a 280 kcal preload compared with well-nourished older and younger women [45]. Further research on under and well-nourished older adults is needed to identify and address factors that contribute to the anorexia of aging.

While aging is not the primary cause of diminished glucose tolerance per se, it affects a large proportion of older adults [46,47]. In the present study, glycemic responses after all caloric treatments returned to, or below, baseline between 90–120 min. Although older adults have shown heightened sensitivity to carbohydrates on oral glucose tolerance tests (OGTT) using 75 g of glucose solution [47,48], our findings suggest glycemic response to carbohydrates may be better regulated in a food form consumed with other macronutrients (i.e., fats and protein). Some studies have suggested that diminished glucose tolerance may be related to reduced insulin sensitivity with aging [47]. However, factors associated with diminished insulin sensitivity with aging are multifactorial, including increased body fat mass, decreased physical activity, increased oxidative stress and inflammation, and sarcopenia [3,49].

While glycemic response did not correlate with FI after individual caloric treatments in this study, a previous study in children suggests an inverse association between glycemic response and FI [17]. Despite the different cooking methods changing the starch structure of foods [11,50] to potentially alter post-prandial glycemic response in children [34] and young adults [19], the lack of difference in glycemic response in the present study may be related to the similar composition of available carbohydrate and fat content of the test treatments. Dietary fat can attenuate glycemic response [51,52] and increase satiety [53] through delayed gastric emptying and starch degradation. In older adults, the effect of fat on gastric emptying may be exacerbated due to rapid antral filling and impaired relaxation of the stomach caused by aging [3,4,5,49]. The effect of macronutrient composition on glycemic and FI regulation in older adults merits further investigation.

Although we examined the effects of commonly consumed forms of white potatoes on satiety, short-term FI, and glycemic response in healthy older adults, there are some limitations to the present study. First, we did not measure gastrointestinal hormones. Older adults have shown to have higher fasting and post-prandial CCK [43] and lower fasting ghrelin concentrations [54] compared with young adults, which may contribute to delayed gastric emptying, higher feelings of satiety, and lower glucose tolerance [55,56]. Examining the gastrointestinal hormone responses in future studies can help elucidate the physiological mechanism of FI and glycemic regulation in older adults. Second, we recruited healthy older adults with no self-reported changes in habitual motivation-to-eat. Future studies should focus on older adults with diminished or increased motivation-to-eat to establish whether there are dietary approaches to improve energy intake and balance in these groups. Third, we did not examine the energy expenditure of participants. Resting metabolic rate and diet-induced thermogenesis are thought to decrease with age due to a decline in lean body mass and Na^+^-K^+^ ATPase activity [3,49,57]. Fourth, we did not measure rest of day FI in this study. In children, however, despite lower FI at 3 h following breakfast of mashed potatoes or French fries with eggs compared with a control meal of cereal and white bread, only the breakfast of French fries with eggs resulted in lower total daily FI [17]. Lastly, it is unknown if short-term FI translates into long-term changes in energy balance. Although a recent study examined the effect of daily consumption of non-fried potatoes for four weeks in young adults [15], the effect in older adults has not been examined. Longitudinal studies are needed to examine the long-term effect of white potatoes on FI regulation and body weight and composition in older adults.

## 5. Conclusions

In conclusion, white potatoes suppressed subjective appetite and lunch FI compared with meal skipping, suggesting white potatoes do not bypass regulatory control mechanisms of energy intake in healthy older adults. Future studies are needed to assess the longer-term effects of consuming white potatoes on energy intake and balance.

## Figures and Tables

**Table 1:** Macronutrient composition of test treatments.

	White Bread	Baked Potatoes	Mashed Potatoes	French Fries
Total energy (kcal)	280	288	283	280
Total protein (g) ^†^	5.7 (8.1%)	4.6 (6.4%)	3.3 (4.7%)	3.0 (4.3%)
Total carbohydrates (g)	35.0	37.1	36.4	36.2
Fiber (g)	1.9	4.0	3.3	3.1
Available carbohydrates (g) ^†^	33.1 (47.3%)	33.1 (46.0%)	33.1 (46.8%)	33.1 (47.3%)
Total fat (g) ^†^	13.7 (44.0%)	13.7 (42.8%)	13.7 (43.6%)	13.7 (44.0%)

Macronutrient composition for French fries was determined by Maxxam Analytics Inc. using the Association of Analytical Chemists method to account for any changes in the fat and fiber content following deep-frying in oil [30]. Macronutrient composition for all other test treatments was calculated based on the United States Department of Agriculture’s Nutrient Database [28] and nutrition information provided by the manufacturer. ^†^ Energy contribution for each macronutrient is shown as a percentage in parentheses.

**Table 2:** Baseline Characteristics.

Variable	Means ± SEM
Age (y)	70.4 ± 0.6
Body mass (kg)	67.9 ± 2.8
Height (cm)	167.2 ± 2.4
BMI (kg/m^2^)	24.1 ± 0.6
Fat mass ^1^ (%)	33.0 ± 2.4
Fat-free mass ^1^ (%)	67.0 ± 2.4
Fasting blood glucose (mmol/L)	4.7 ± 0.1

All values are presented as means ± SEM, *n* = 20 (7 males and 13 females). BMI = body mass index. ^1^ Body composition measures (i.e., fat mass and fat-free mass) were estimated using the Bod Pod [26].

**Table 3:** Effect of treatment on food intake (FI), water intake, and pleasantness of test treatment.

	Test Treatment					
	Meal Skipping	White Bread	Baked Potatoes	Mashed Potatoes	French Fries	*p*-Value
Lunch FI (kcal)	951.2 ± 122.6 ^a^	776.9 ± 69.8 ^b^	736.1 ± 76.4 ^b^	771.6 ± 89.4 ^b^	755.8 ± 100 ^b^	<0.001
Cumulative FI ^1^ (kcal)	951.2 ± 122.6	1057.4±69.8	1024.1±76.4	1035.8±89.4	1035.8±100	0.26
Water intake with test treatment (g)	319.2 ± 39.1	375.9 ± 28.2	350.3 ± 32.4	368.3 ± 41.5	340.2 ± 33.9	0.60
Water intake at lunch (g)	352 ± 31.4	382.7 ± 38.7	330.9 ± 27.3	390.9 ± 29.4	356.9 ± 37.0	0.27
Pleasantness of test treatment (/100 units)	-	42.2 ± 6.5 ^a^	60.1 ± 5.6 ^b^	49.1 ± 7.2 ^a,b^	61.5 ± 5.6 ^b^	<0.001

All values are means ± SEM, *n* = 20. FI = food intake. Different letters in each row represent significant differences (*p* < 0.05) by one-factor ANOVA with Tukey–Kramer’s post hoc test to account for multiple comparisons. ^1^ Cumulative FI = test treatment + lunch FI (ad libitum pizza lunch).
